# Investigating the Demographic Foundation of Fertility: Temporal Trends in the Female Population of Reproductive Age in Greece, 1956–2024

**DOI:** 10.7759/cureus.110917

**Published:** 2026-06-15

**Authors:** Nikolaos Vlachadis, Chrysi Christodoulaki, Charalampos Theofanakis, Nikolaos Machairiotis, Rafaela Panagopoulou, Ioanna Petrakou, Alexios Kozonis, Sofoklis Stavros, Periklis Panagopoulos

**Affiliations:** 1 Third Department of Obstetrics and Gynecology, School of Medicine, National and Kapodistrian University of Athens, Attiko University Hospital, Athens, GRC; 2 Department of Obstetrics and Gynecology, General Hospital of Chania, Chania, GRC; 3 Postgraduate Program "High-Risk Pregnancy", School of Medicine, National and Kapodistrian University of Athens, Attiko University Hospital, Athens, GRC

**Keywords:** fertility, fertility rates, greece, population, temporal trends, women of childbearing age, women of reproductive age

## Abstract

Objective: The objective of this study is to investigate long-term temporal trends in the size and age structure of the population of women of reproductive age (WRA) in Greece.

Materials and methods: Data on the estimated mid-year WRA population (15-44 years) in Greece were obtained from the Hellenic Statistical Authority for the period 1956-2024. For each year, the percentage distribution of the WRA population across five-year age groups was calculated. Temporal trends were assessed using Joinpoint regression analysis, and annual percent changes (APCs) with corresponding 95% confidence intervals (95% CIs) were estimated.

Results: The WRA population in Greece fluctuated without a significant overall trend between 1956 and 1974, reaching 2,026,378 in 1968 and declining to 1,883,459 in 1974. Subsequently, it increased by 25% to a historic peak of 2,353,200 in 2000, with significant upward trends during 1974-1987 (APC = 0.7, 95% CI: 0.1 to 1.4) and 1987-1995 (APC = 1.3, 95% CI: 0.3 to 2.0). Since 2000, the WRA population has declined by 28%, reaching a historic low of 1,701,390 in 2024. The decline was modest during 2000-2010 (APC = −0.5, 95% CI: −0.5 to −0.4) but accelerated thereafter, with the WRA population decreasing by 24% between 2010 and 2024 at an almost exponential annual rate of 2.0%. Age-specific analysis revealed progressive aging of the WRA population. The modal age group shifted from 15-19 years during 1975-1988 to 40-44 years since 2010, highlighting a marked shift toward older reproductive ages.

Conclusions: The WRA population in Greece entered a period of marked decline after reaching a historic peak in 2000, with an almost exponential decrease observed after 2010. Greece lost nearly one-quarter of its female population of childbearing age between 2010 and 2024. This demographic contraction was accompanied by pronounced aging of the WRA population, developments that are likely to substantially constrain the future annual number of births in the country.

## Introduction

The study of fertility rates is a fundamental component of human reproduction research. Accurate assessment of fertility trends, together with changes in the population size and age structure, is essential for anticipating future social, economic, and public health challenges and for guiding healthcare and family-planning policies [1,2].

Over the past several decades, profound changes in reproductive behavior have been observed worldwide. Estimates indicate that the global total fertility rate (TFR) declined by more than half between 1950 and 2021, from approximately 4.8 to 2.2 live births per woman. During the same period, the annual number of live births worldwide increased from about 93 million in 1950 to a peak of 142 million in 2016, before declining to 129 million in 2021. Fertility rates decreased in virtually all countries, and the majority have now entered a phase of sub-replacement fertility, defined as a TFR below 2.1 live births per woman, the conventional threshold for population replacement [1].

These profound changes in fertility patterns have contributed to the demographic transition, whereby populations shift from a regime of high fertility and high mortality, characterized by a predominantly young age structure, to one of low fertility and low mortality, accompanied by progressive population aging. Over time, persistently low fertility rates may lead to inverted population pyramids, characterized by a growing proportion of older individuals and a shrinking population of women of reproductive age (WRA; aged 15-44 years) [1,3].

A wide range of factors have been implicated in the fertility decline observed in modern developed societies. These include urbanization, higher levels of female education and workforce participation, changing social norms regarding marriage and family life, declining religiosity, socioeconomic constraints, postponement of childbearing, and biological factors such as obesity and exposure to environmental pollutants. In addition, increased access to and use of effective contraceptive methods have played a major role in shaping contemporary fertility patterns [2,4-6].

Although considerable attention has been devoted to identifying and studying the factors that influence contemporary human fertility, the role of the WRA population is often overlooked. Yet, the size and age composition of the WRA population constitute an equally important determinant of the number of births in a country. Indeed, Europe and many other developed regions have entered a critical stage of demographic transition, characterized by the establishment of negative demographic momentum. This phenomenon translates into declining numbers of births regardless of small annual fluctuations in fertility rates, owing to the progressive shrinkage of the WRA population resulting from low birth rates in previous decades [7]. This issue appears particularly relevant to Greece, which has remained below replacement-level fertility for more than four decades and has witnessed a dramatic decline in birth numbers over the same period [8,9]. Therefore, the aim of the present study was to investigate and analyze the long-term temporal trends in both the size and age composition of the WRA population in Greece. By doing so, the study seeks to highlight the importance of this often-overlooked demographic determinant of fertility and to provide a basis for further research into its contribution to the evolution of birth trends in the country.

## Materials and methods

Study population and parameters

This study used official mid-year population estimates obtained from the Hellenic Statistical Authority (ELSTAT) [10]. The dataset consisted of the estimated mid-year WRA population, defined as women aged 15-44 completed years, in Greece. For each year between 1956 and 2024, the percentage distribution of the WRA population across the following age groups was calculated relative to the total WRA population of the corresponding year: 15-19, 20-24, 25-29, 30-34, 35-39, and 40-44 years. In addition, annual data on incoming and outgoing migrant women aged 15-44 years were obtained from the Hellenic Statistical Authority for the period 2010-2024. The annual net migration balance of the WRA population was calculated as the difference between the number of immigrant and emigrant women for each year.

Statistical analysis

Data analysis was performed using Microsoft Excel 2010 (Microsoft Corporation, Redmond, WA, USA). Temporal trends were assessed using the Joinpoint Regression Program, version 6.0.0 (National Cancer Institute, Bethesda, MD, USA), which identifies joinpoints corresponding to statistically significant changes in trends using a log-linear model. A maximum of six joinpoints was allowed in the regression models. The selection of joinpoints was based on Monte Carlo permutation tests. The analyses assumed homoscedastic errors and no autocorrelation. For each segment between two joinpoints, the annual percent change (APC) and corresponding 95% confidence intervals (95% CIs) were calculated. Additionally, the average annual percent change (AAPC) was calculated to summarize overall temporal trends across multiple segments. The statistical significance of APC and AAPC estimates was assessed using two-sided t-tests to determine whether the slope differed significantly from zero, with p < 0.05 considered statistically significant.

Given the long duration of the study period (1956-2024) and to better characterize temporal changes in the WRA population, the trend analysis was divided into two phases. The first phase extended from 1956 to 2000, corresponding to the historical peak of the WRA population in Greece, whereas the second phase covered the period from 2000 to 2024. Recent trends in the WRA population during 2000-2024 were further examined using an exponential regression model, and the coefficient of determination (R²) was calculated to assess goodness of fit.

## Results

The WRA population in Greece was 1,953,696 in 1956. Following a modest increase to 2,026,378 in 1968 (4% increase), the population declined to a minimum of 1,883,459 in 1974, corresponding to a 7% decrease between 1968 and 1974. This was followed by a prolonged period of increase, reaching a historic peak of 2,353,200 in 2000, representing a 25% increase compared with 1974. Temporal trends were not statistically significant during 1956-1974. Subsequently, the WRA population increased during 1974-1987 (APC = 0.7, 95% CI: 0.1 to 1.4), with a more accelerated rise during 1987-1995 (APC = 1.3, 95% CI: 0.3 to 2.0), whereas the upward trend was no longer statistically significant thereafter until 2000. Overall, the WRA population increased between 1974 and 2000 at an average annual rate of 0.9% (95% CI: 0.8% to 0.9%, p < 0.001).

**Figure 1:**
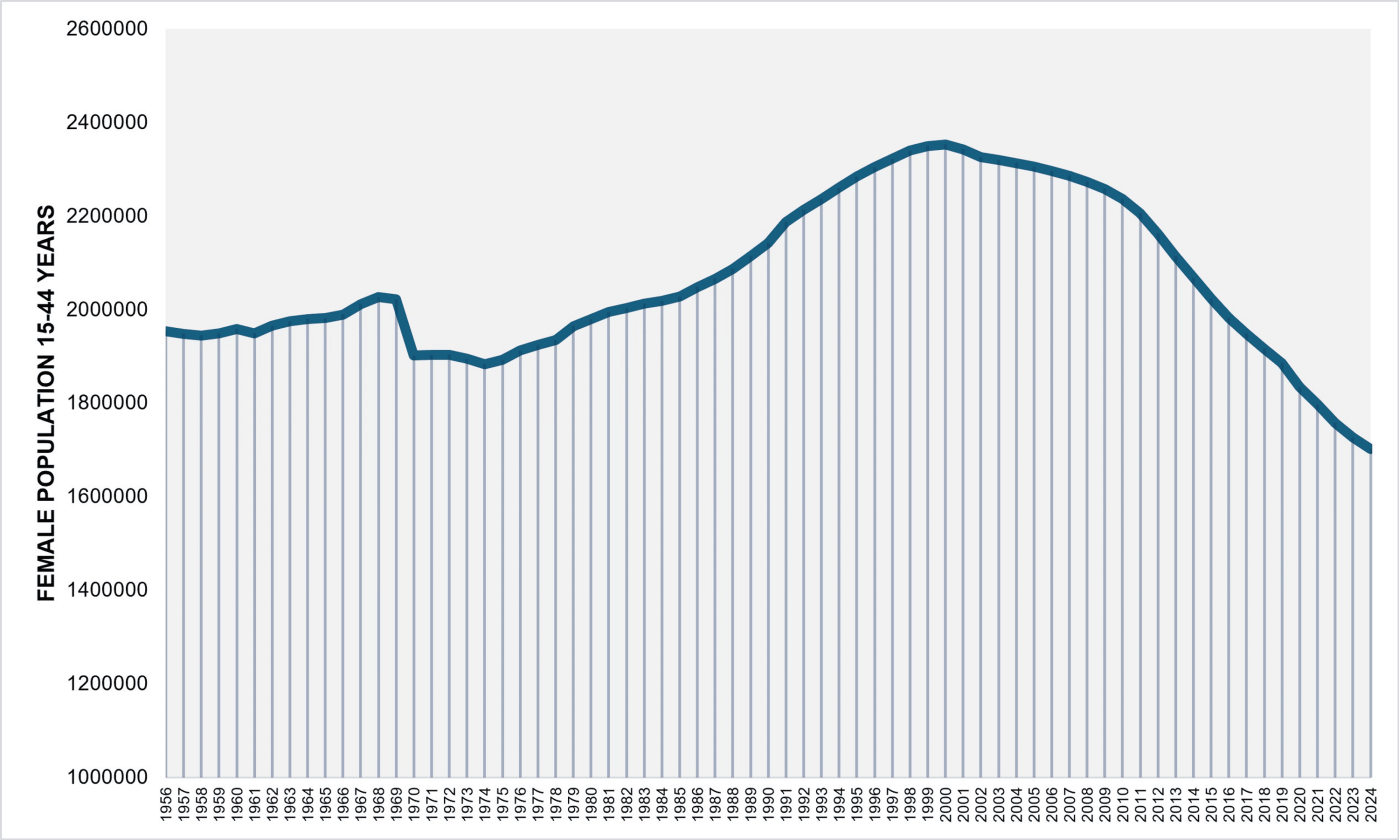
Population of women of reproductive age (15-44 years) in Greece, 1956-2024

**Figure 2:**
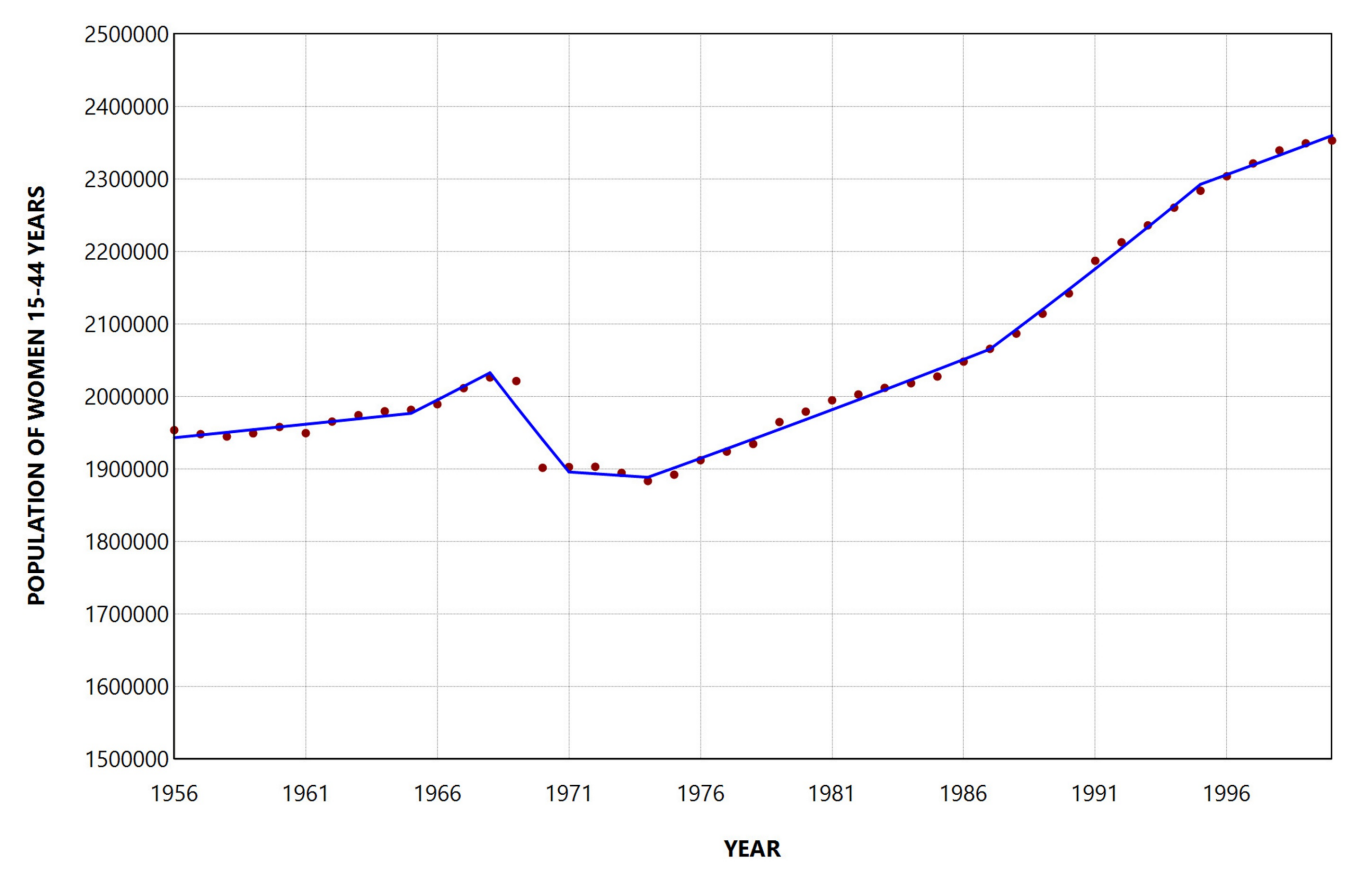
Trends in the population of women of reproductive age (15-44 years) in Greece, 1956-2000

**Figure 3:**
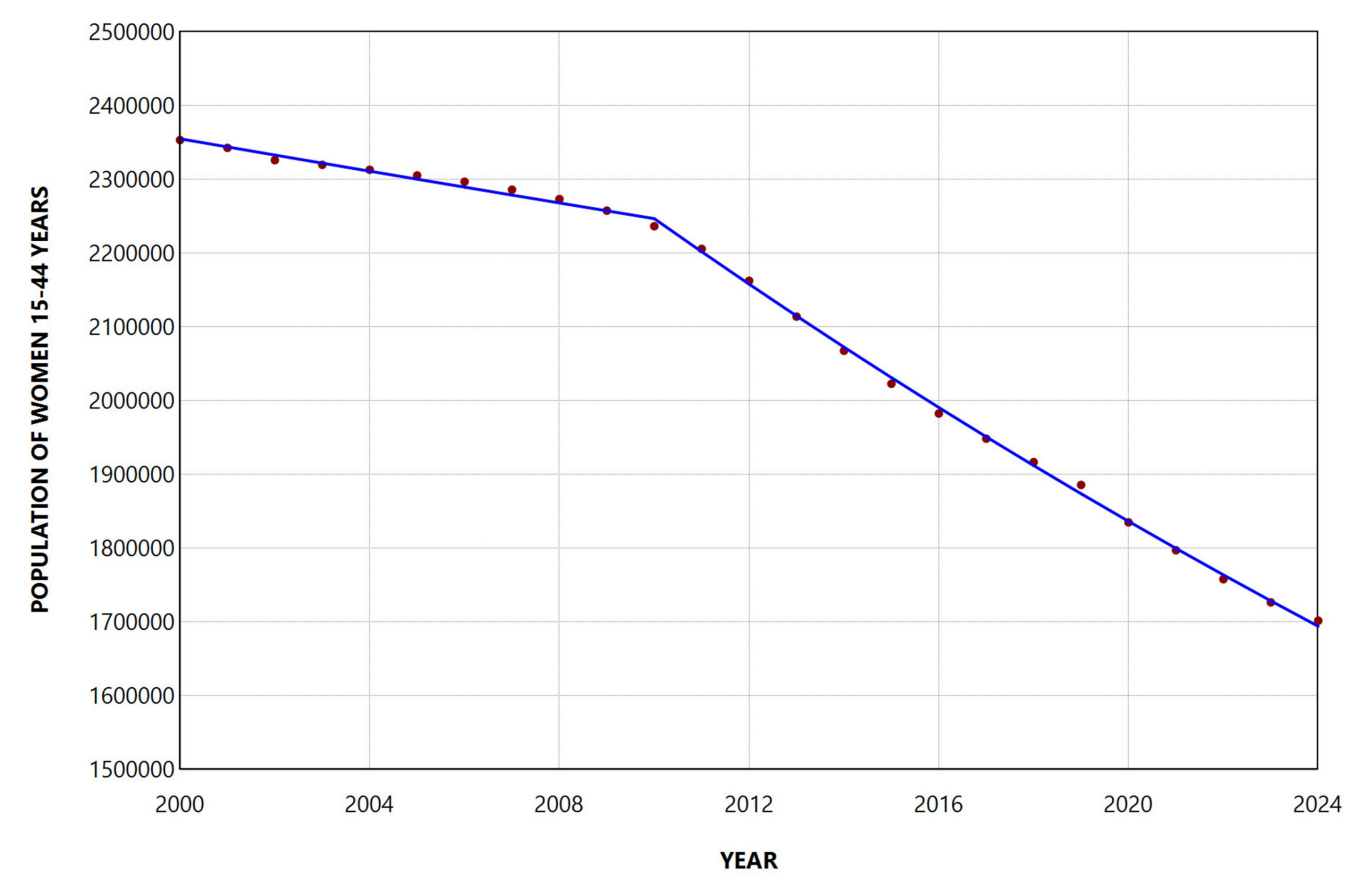
Trends in the population of women of reproductive age (15-44 years) in Greece, 2000-2024

**Figure 4:**
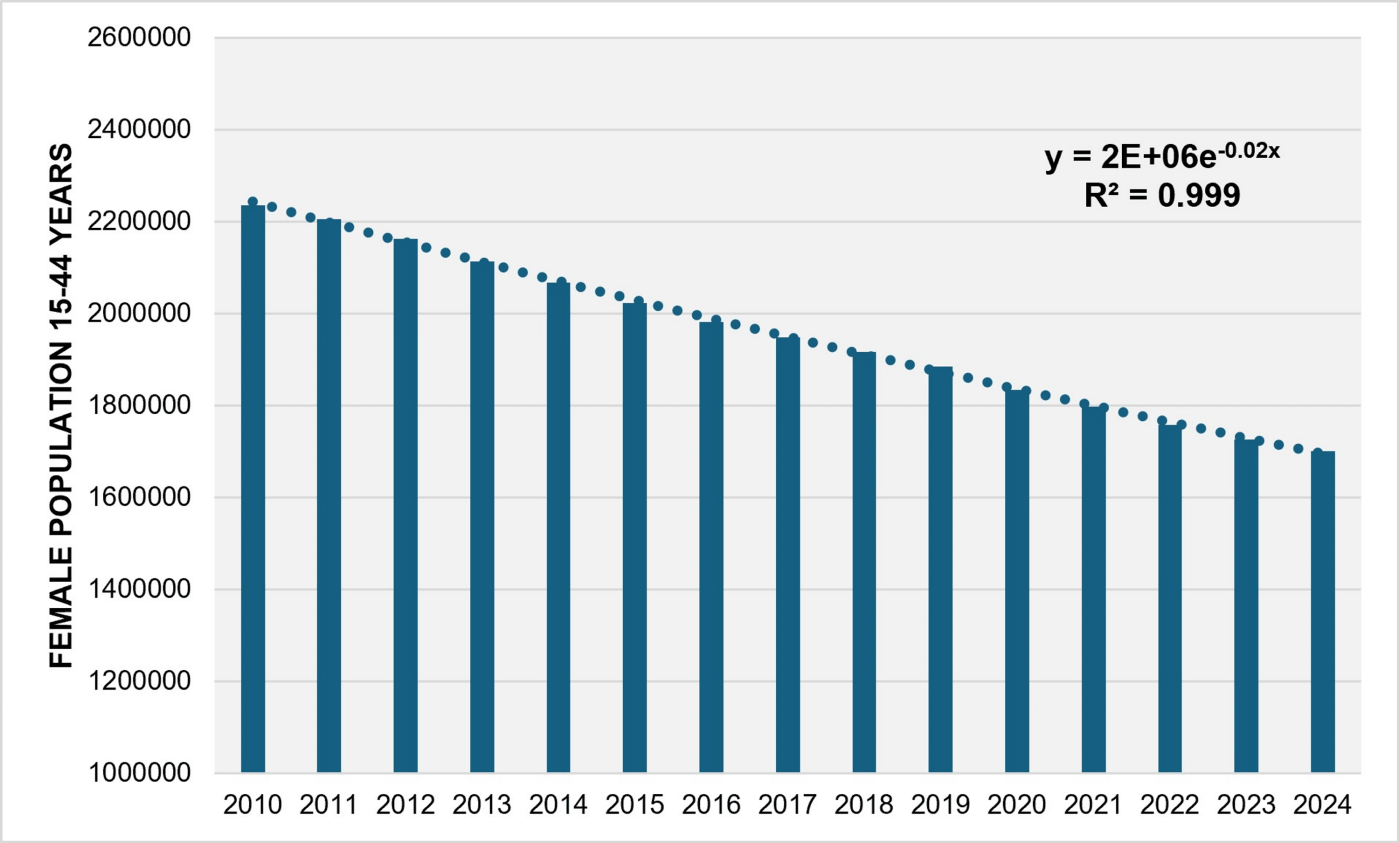
Exponential model fit for the decline of the population of women of reproductive age (15-44 years) in Greece, 2010–2024 R-squared (R²): coefficient of determination

Since 2000, the WRA population in Greece has declined by 28%, reaching a historic low of 1,701,390 in 2024. The downward trend was relatively modest during the first decade (2000-2010: APC = −0.5, 95% CI: −0.5 to −0.4) and became substantially steeper during 2010-2024 (APC = −2.0, 95% CI: −2.0 to −2.0). Further analysis demonstrated that the decline in the WRA population between 2010 and 2024 followed an almost perfectly exponential pattern (R² = 0.999). During 2010-2024, the WRA population declined by 534,976 women, corresponding to a relative decrease of 24% (Figures 1-4, Tables 1, 2).

**Table 1:** Trends in the population of women of reproductive age (15-44 years) in Greece, 1956-2000 *Statistically significant

Segment	Annual percent change	95% confidence interval	P-value
1956-1965	0.2	-0.5 to 0.9	0.294
1965-1968	0.9	-2.0 to 1.3	0.286
1968-1971	-2.3	-2.8 to 1.1	0.226
1971-1974	-0.1	-2.1 to 1.3	0.972
1974-1987	0.7	0.1 to 1.4	0.031*
1987-1995	1.3	0.3 to 2.0	0.022*
1995-2000	0.6	-0.3 to 1.1	0.092

**Table 2:** Trends in the population of women of reproductive age (15-44 years) in Greece, 2000-2024 *Statistically significant

Segment	Annual percent change	95% confidence interval	P-value
2000-2010	-0.5	-0.5 to -0.4	< 0.001*
2010-2024	-2.0	-2.0 to -2.0	< 0.001*

**Figure 5:**
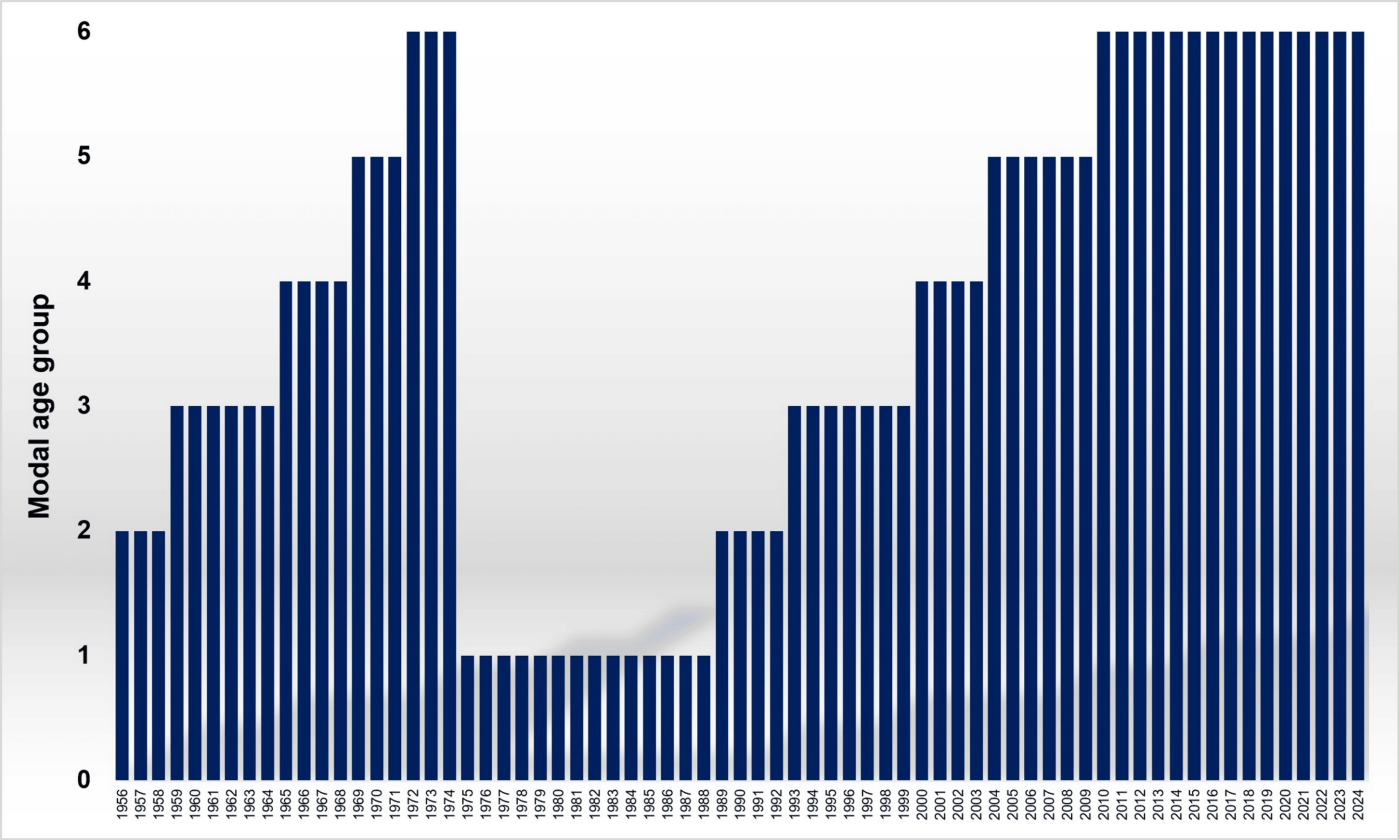
Modal age group of the population of women of reproductive age (15-44 years) in Greece, 1956-2024 Age groups of the population of women of reproductive age: 1: 15-19 years, 2: 20-24 years, 3: 25-29 years, 4: 30-34 years, 5: 35-39 years, 6: 40-44 years

In the age-specific analysis of the WRA population in Greece, the predominant age group showed an upward shift between 1956 and 1974. Specifically, the modal age group was 20-24 years during 1956-1958, 25-29 years during 1959-1964, 30-34 years during 1965-1968, 35-39 years during 1969-1971, and 40-44 years during 1972-1974. In 1975, the modal age group shifted back to 15-19 years and remained so until 1988. Thereafter, the same upward age-shift pattern re-emerged and continued through 2024. The modal age group was 20-24 years during 1989-1992, 25-29 years during 1993-1999, 30-34 years during 2000-2003, and 35-39 years during 2004-2009, whereas since 2010 the modal age group of the WRA population has been 40-44 years (Figure 5).

During the recent period of decline in the WRA population in Greece, decreasing trends were observed across all age groups. However, the decline was more pronounced in the 20-34 age range. Specifically, the decrease occurred with an AAPC of -1.9 (95% CI: -2.0 to -1.9, p < 0.001) for the 20-24 age group, -2.2 (95% CI: -2.3 to -2.2, p < 0.001) for the 25-29 age group, and -2.0 (95% CI: -2.1 to -2.0, p < 0.001) for the 30-34 age group. Between 2000 and 2024, the WRA population decreased by 37% in the 20-24 age group, 42% for the 25-29 age group, and 39% for the 30-34 age group. The 20-24 age group reached a historical high in 1998 (405,019) and its lowest value in 2022 (249,580), followed by a slight increase by 2024 (253,465). The 25-29 age group peaked in 2000 (422,230) and reached its period low in 2024 (245,326). Lastly, the 30-34 age group recorded a high of 427,785 in 2001 and declined to its lowest recorded value in 2024 (261,988).

**Figure 6:**
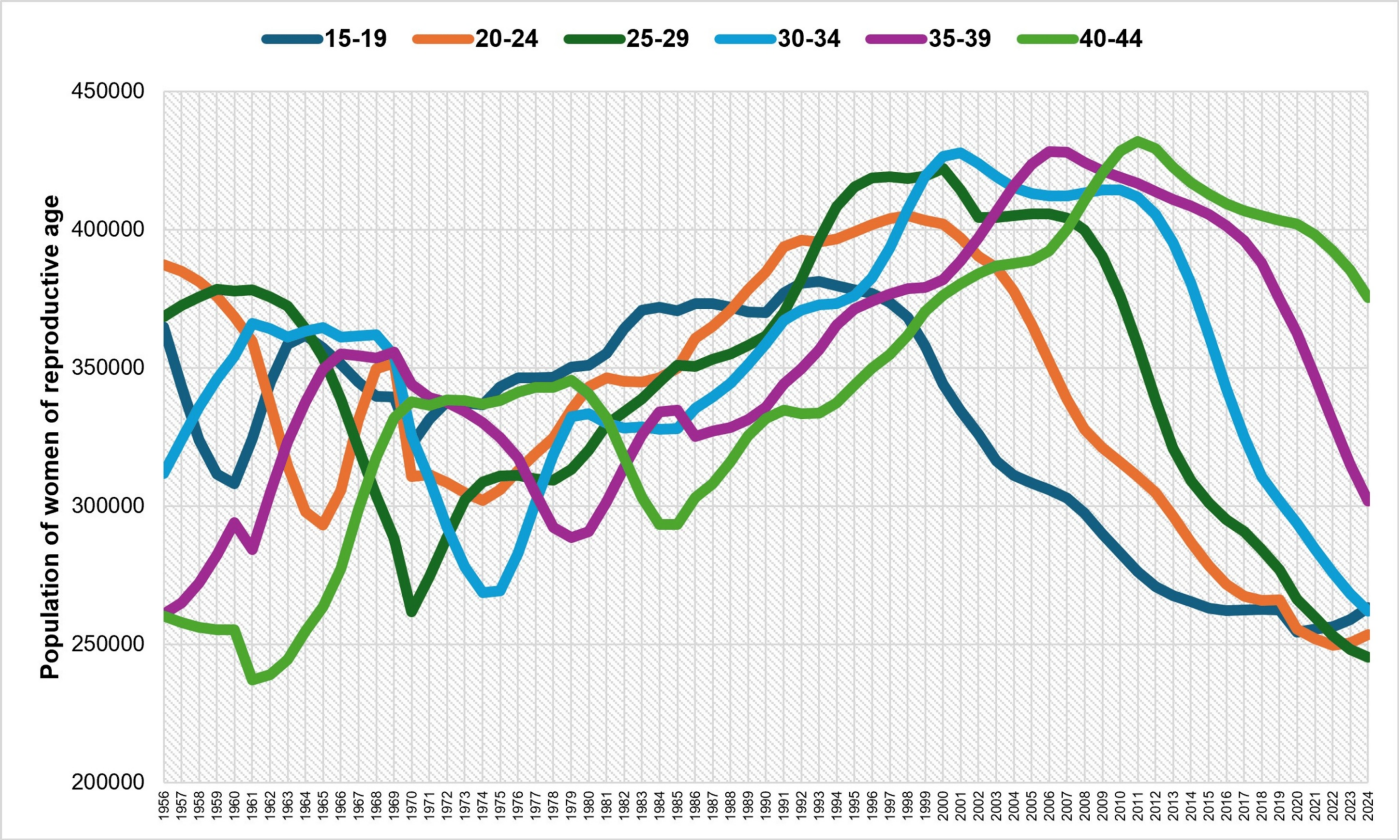
Population of women of reproductive age (15-44 years) by age group in Greece, 1956-2024

The decline was less pronounced in the 15-19 age group (AAPC = -1.1, 95% CI: -1.2 to -1.1, p < 0.001) and the 35-39 age group (AAPC = -1.0, 95% CI: -1.0 to -1.0, p < 0.001). The adolescent age group (15-19 years) peaked in 1993 (381,171 women) and reached its period low in 2020 (254,402), followed by a slight recovery by 2024, reaching 263,389 women. The 35-39 age group recorded a high of 428,158 in 2006, followed by a subsequent decline through 2024 (301,877). The lowest value of the period for the 35-39 age group was observed in 1956 (260,990). From 2000 to 2024, the decrease was 23% for adolescents (15-19 years) and 21% for the 35-39 years age group. For the oldest age group (40-44 years), no overall trend was observed during 2000-2024 (AAPC = −0.0, 95% CI: −0.1 to 0.0, p = 0.436). The population of women aged 40-44 years increased from 376,355 in 2000 to an all-time peak of 431,921 in 2011, before declining to 375,345 in 2024. The lowest recorded population size for this age group was 237,107 in 1961 (Figure 6).

In all age groups and time segments, the trends were generally downward. The exceptions were rising trends for ages 35-39 and 40-44 during 2000-2006 (APC = 2.0; 95% CI, 1.9-2.1 and APC = 0.7; 95% CI, 0.4-0.9, respectively) and for ages 15-19 and 20-24 during 2022-2024 (APC = 1.6; 95% CI, 0.6-2.2 and APC = 1.1; 95% CI, 0.4-1.7, respectively). Additionally, the trends appeared stable for the 25-29 age group between 2002 and 2006 and the 30-34 age group between 2006 and 2010 (Table 3).

**Table 3:** Trends in the population of women of reproductive age (15-44 years) by age group in Greece, 2000-2024 *Statistically significant

Age (years)	Segment	Annual percent change	95% confidence interval	P-value
15-19	2000-2003:	-2.8	-3.7 to -2.2	< 0.001*
	2003-2008	-1.2	-1.4 to -0.5	< 0.001*
	2008-2012	-2.5	-3.1 to -2.1	< 0.001*
	2012-2022	-0.5	-0.7 to -0.4	< 0.001*
	2022-2024	1.6	0.6 to 2.2	0.004*
20-24	2000-2004	-1.5	-1.8 to -1.1	< 0.001*
	2004-2008	-3.6	-4.1 to -3.2	< 0.001*
	2008-2012	-1.7	-2.1 to -1.2	< 0.001*
	2012-2016	-2.9	-3.4 to -2.7	< 0.001*
	2016-2019	-0.9	-1.3 to -0.6	< 0.001*
	2019-2022	-2.0	-2.4 to -1.6	< 0.001*
	2022-2024	1.1	0.4 to 1.7	0.002*
25-29	2000-2002	-2.2	-2.8 to -1.4	< 0.001*
	2002-2006	0.2	-0.1 to 0.7	0.119
	2006-2009	-1.1	-1.7 to -0.7	< 0.001*
	2009-2014	-4.8	-5.0 to -4.6	< 0.001*
	2014-2018	-1.9	-2.2 to -1.6	< 0.001*
	2018-2022	-3.0	-3.4 to -2.7	< 0.001*
	2022-2024	-1.4	-2.2 to -0.9	< 0.001*
30-34	2000-2006	-0.7	-1.3 to -0.5	< 0.001*
	2006-2010	0.3	-0.0 to 0.8	0.080
	2010-2013	-1.4	-2.7 to -0.9	< 0.001*
	2013-2018	-4.9	-5.3 to -4.7	< 0.001*
	2018-2024	-2.8	-3.0 to -2.5	< 0.001*
35-39	2000-2006	2.0	1.9 to 2.1	< 0.001*
	2006-2017	-0.7	-0.7 to -0.7	< 0.001*
	2017-2020	-3.1	-3.4 to -2.9	< 0.001*
	2020-2024	-4.6	-4.8 to -4.4	< 0.001*
40-44	2000-2006	0.7	0.4 to 0.9	0.002*
	2006-2011	2.0	1.8 to 2.5	< 0.001*
	2011-2015	-1.4	-1.8 to -1.1	0.002*
	2015-2021	-0.5	-0.7 to -0.0	0.045*
	2021-2024	-1.9	-2.5 to -1.5	< 0.001*

The proportion of women aged 15-19 and 20-24 years among the total WRA population peaked in 1956 (18.7% and 19.8%, respectively) and generally declined thereafter, reaching their lowest values of 12.5% in 2011-2012 and 13.7% in 2016-2017, respectively. The proportions of women aged 25-29 and 30-34 years reached their highest levels in 1959 (19.4%) and 1961 (18.8%), respectively, whereas their lowest values were recorded in 1970 (13.8%) and 1975 (14.2%), respectively. In contrast, the proportions of women aged 35-39 and 40-44 years increased substantially over time, rising from 13.4% in 1956 to 20.3% in 2017, and from 12.2% in 1961-1962 to 22.3% in 2022-2023, respectively.

**Figure 7:**
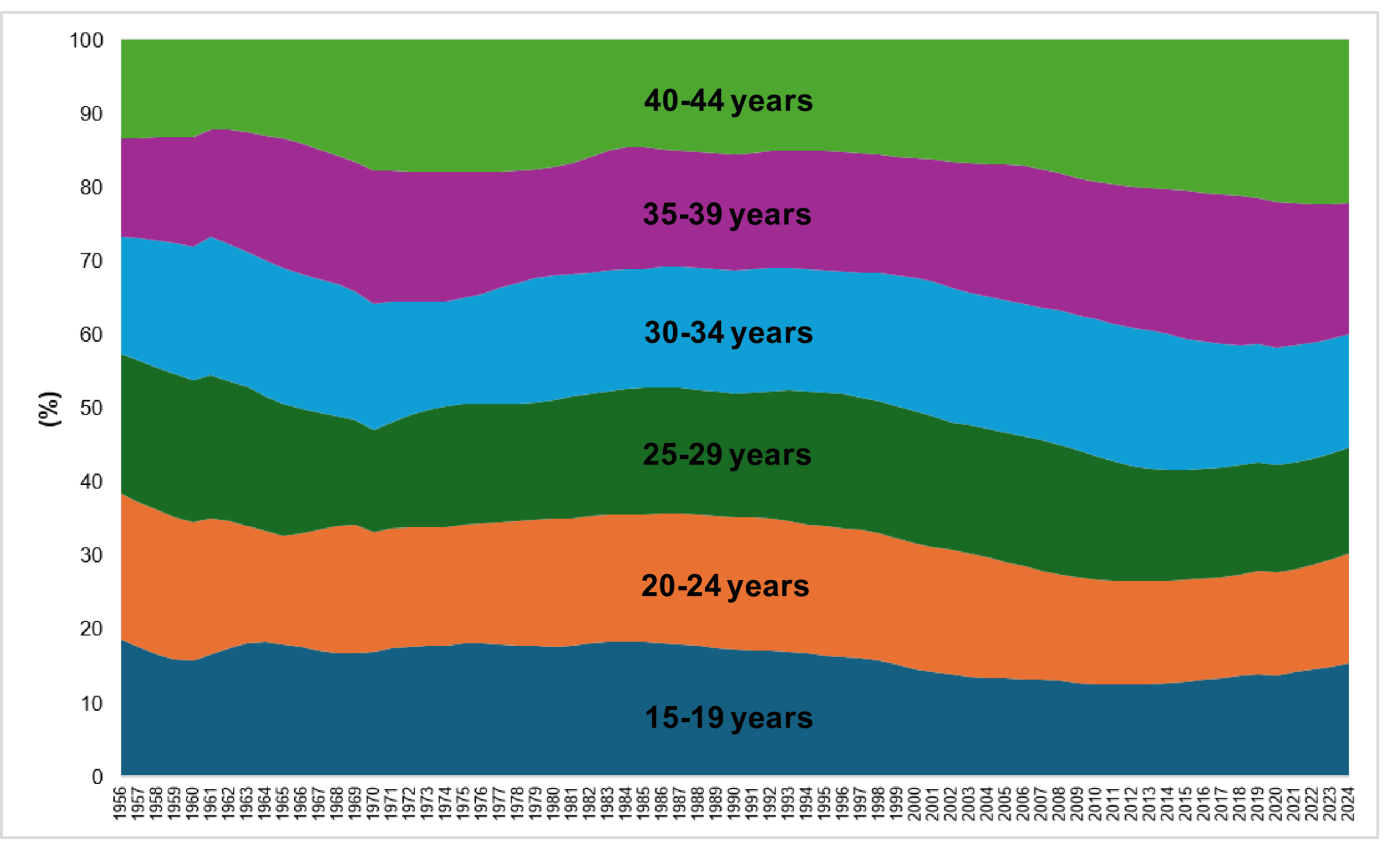
Percentage distribution of the population of women of reproductive age (15–44 years) by age group in Greece, 1956–2024

During 2000-2024, the decline in the size of the WRA population in Greece was accompanied by relative aging within the reproductive-age female population, characterized by an increasing proportion of women aged 35 years and older. The proportion of women aged 35-39 and 40-44 years among the total WRA population increased from 16.2% and 16.0% in 2000 to 17.7% and 22.1% in 2024, respectively. In contrast, the proportions of women aged 20-24, 25-29, and 30-34 years declined from 17.1% to 14.9%, from 17.9% to 14.4%, and from 18.1% to 15.4%, respectively. The only exception was the adolescent age group (15-19 years), which showed a slight relative increase from 14.6% to 15.5%. Overall, the proportion of women aged 35-44 years among the WRA population increased from 32.2% in 2000 to 39.8% in 2024, reaching an intermediate peak of 41.7% in 2020, the highest value recorded since 1956. Importantly, since 2009, the 35-39- and 40-44-year age groups have been the most prevalent age groups within the WRA population (Figure 7).

Between 2010 and 2024, the migration balance among women of childbearing age (15-44 years) in Greece remained negative throughout the study period. The deficit peaked during 2012-2015, subsequently moderated, increased again in 2021, and then declined markedly thereafter (Table 4).

**Table 4:** Immigration, emigration, and net migration balance among women of reproductive age (15–44 years) in Greece, 2010–2023

Year	Female immigrants aged 15–44 years	Female emigrants aged 15–44 years	Net migration balance of women aged 15–44 years
2010	17564	21041	-3,477
2011	17575	27379	-9,804
2012	17024	33463	-16,439
2013	16947	31826	-14,879
2014	15707	27533	-11,826
2015	16795	30736	-13,941
2016	25239	32298	-7,059
2017	24668	31667	-6,999
2018	25981	32058	-6,077
2019	27247	30177	-2,930
2020	21386	25814	-4,428
2021	13718	29314	-15,596
2022	25317	31691	-6,374
2023	28358	30142	-1,784

## Discussion

In this nationwide demographic analysis spanning nearly seven decades, substantial changes were observed in both the size and age structure of the WRA population in Greece. After a prolonged period of relative stability between 1956 and 1974 and a gradual increase between 1974 and 2000, the WRA population entered a phase of marked decline, reaching its lowest recorded level in 2024. Importantly, this reduction was accompanied by substantial changes in the age structure of the WRA population and pronounced aging among women of childbearing age in the country.

After several fluctuations, the WRA population in Greece reached a low point in 1974. This was followed by a continuous upward trend, increasing on average by 0.9% annually and reaching a historic peak in 2000. The overall 25% increase in the WRA population between 1974 and 2000 likely reflected the entry into reproductive age of the large birth cohorts born during the postwar decades (1950s-1970s). Moreover, this increase was further reinforced by the positive migration balance recorded in Greece during the second half of the 1970s and throughout the 1980s and 1990s.

However, since 2000, the female fertile population in Greece has deteriorated dramatically, declining by 28% between 2000 and 2024. The downward trend was relatively modest during 2000-2010, with an annual decrease of 0.5%, but accelerated markedly during 2010-2024, to a 2% annual decline. Overall, nearly one in four women of childbearing age was lost from the country between 2010 and 2024. Particularly concerning was the almost perfectly exponential decline observed after 2010, suggesting a persistent and accelerating demographic contraction rather than a temporary fluctuation. This major reduction in the WRA population primarily reflects a cohort-replacement mechanism, whereby the large female birth cohorts born during the 1960s progressively aged out of the reproductive-age range and were replaced by smaller cohorts born during subsequent decades of declining births [8]. Importantly, the birth-cohort deficit was further aggravated by the negative migration balance observed in the country. This likely reflected initially the severe economic recession following the implementation of the economic adjustment programs imposed in Greece and, more recently, the renewed socioeconomic deterioration observed during the post-COVID-19 period.

The decline was not evenly distributed across age groups. Women aged 20-34 years, who represent the biologically core reproductive ages, experienced the steepest reductions. Between 2000 and 2024, the populations aged 20-24, 25-29, and 30-34 years decreased by 37%, 42%, and 39%, respectively. In contrast, the 35-39 years age group saw a smaller decrease of 21%, similar to that in adolescents (15-19 years, -23%), whereas the oldest reproductive age group (40-44 years) remained essentially unchanged.

The aging of the WRA population has been observed as early as the 1980s, with the progressive predominance of increasingly older age groups, shifting successively from the 15-19 age group to the older 35-39 age group (2004-2009). Since 2010, women aged 40-44 years have constituted the modal age group of the WRA population in Greece. These findings are consistent with a previous report demonstrating increasing maternal age in Greece, with the most pronounced relative increase observed among mothers aged ≥ 40 years [11]. The decline in the number of women of childbearing age during 2000-2024 was accompanied by an increasing proportional representation of women aged ≥ 35 years within the WRA population. Specifically, the proportion of women aged 35-44 years increased from approximately one-third of the WRA population in 2000 to almost 40% in 2024, reaching a peak of 41.7% in 2020. These findings indicate substantial reproductive population aging in Greece. Importantly, since 2009, the two oldest reproductive age groups (35-39 and 40-44 years) have represented the largest age groups within the WRA population. This demographic shift has important implications for national fertility patterns, as advancing female age is associated with lower natural fecundity, increased use of medically assisted reproduction, and higher rates of adverse obstetric outcomes [12-15].

From a demographic and public health perspective, these findings are particularly important because the WRA population constitutes the demographic foundation of fertility within a country. Greece has experienced a marked decline in births since the 1980s, which was temporarily attenuated during the early 21st century before accelerating again after 2008 [8]. Since then, the country appears to have entered a low-fertility spiral driven by the continuing contraction of the WRA population. In addition, the persistent decline in births, combined with the negative migration balance of young women associated with adverse socioeconomic conditions in the country, has further accelerated aging within the WRA population, as the older birth cohorts remain substantially larger than the younger ones. This shrinking and aging reproductive-age population generates a negative demographic momentum that is particularly difficult to reverse, even in the presence of modest improvements in fertility rates. Consequently, the continuing reduction and progressive aging of the WRA population inevitably constrain future birth potential and create conditions consistent with a low-fertility trap [7].

The present study has several important strengths. It was based on nationwide official population data covering the entire country over a very long observation period (1956-2024), allowing robust assessment of long-term demographic trends. The use of Joinpoint regression analysis enabled the identification of distinct periods during which statistically significant changes in demographic trends occurred. In addition, the separate evaluation of age-specific WRA subgroups provided a more detailed characterization of reproductive population aging in Greece.

Nevertheless, several limitations should be acknowledged. The present study analyzed official national demographic data on the population of women of childbearing age in Greece; however, these figures do not represent exact annual population counts but rather mid-year population estimates. The use of mid-year population estimates is standard in demographic research and fertility analyses because it statistically approximates the population exposed to demographic events, particularly births, during a given year. These estimates are derived from the most recent national census data and are subsequently adjusted according to births, deaths, and net migration. Consequently, some degree of estimation error cannot be excluded. In addition, the present study defined the WRA population as women aged 15-44 years in order to focus on the age range corresponding to natural fertility. This age range is also used in official fertility statistics in the United States published by the Centers for Disease Control and Prevention [16]. Nevertheless, this population does not fully encompass all childbearing women in a given year, as an increasing proportion of births in Greece occurs among women aged 45 years and older. However, natural fertility beyond 45 years of age is extremely limited, and pregnancies at these ages are almost exclusively achieved through medically assisted reproduction, including the use of cryopreserved gametes and donor oocytes [17]. Finally, the present study did not comprehensively investigate the determinants underlying the observed changes in the WRA population, such as historical birth cohort patterns, migration balance, and socioeconomic conditions.

The present study provided a comprehensive analysis of the size, age structure, and temporal trends of the WRA population in Greece over nearly seven decades. Following a period of increase during 1974-2000, Greece has experienced a substantial demographic shrinking of its female population of childbearing age since 2000. The decline became particularly pronounced after 2010, when the WRA population began decreasing at an almost exponential annual rate of 2%, resulting in Greece losing nearly one-quarter of its female reproductive-age population. This decrease was accompanied by parallel aging of the reproductive-age population, characterized by the relative predominance of women aged 35-44 years.

This downward and aging trend largely reflects the decline in births during previous decades and has been further reinforced by the recent negative migration balance in the country. These demographic changes are likely to have major implications for future fertility dynamics in Greece. As the WRA population, together with fertility rates, represents one of the two principal demographic determinants of the number of births in a country, continuous monitoring of reproductive-age population trends, combined with further investigation alongside fertility rates, is essential to further elucidate their contribution to the ongoing decline in births in the country. The close interplay between changes in the WRA population and fertility rates in shaping trends in the number of births has previously been demonstrated in a report of the decline in births during the first four years of the financial recession in Greece (2008-2012), where approximately 40% of the observed reduction in births was attributable to shrinkage of the WRA population [18]. Furthermore, public health and reproductive health policies aimed at preserving reproductive health and optimizing natural fertility among WRA, combined with broader social and state policies supporting family formation and childbearing, appear imperative for sustaining fertility in Greece amid the current low-fertility spiral [19,20].

## Conclusions

The present study demonstrated substantial long-term changes in both the size and age composition of the WRA population in Greece between 1956 and 2024. After increasing during 1974-2000, the WRA population entered a period of marked decline after 2000, with an almost exponential decrease observed after 2010. This decline was accompanied by aging of the reproductive-age population, characterized by the predominance of older reproductive-age groups (35-44 years). Amid negative demographic momentum and a fertility trap, integrated family-planning, reproductive health, and pro-natal policies appear imperative.
